# Differences in quality of life and fitness level among men and women in the adulthood: a cross-sectional analysis

**DOI:** 10.1186/s13690-024-01244-1

**Published:** 2024-01-29

**Authors:** Celia Alvarez-Bueno, Alicia del Saz-Lara, Ivan Cavero-Redondo, Eva Rodriguez-Gutierrez, Marta Gonzalez-Molinero, Bruno Bizzozero-Peroni, Carlos Pascual-Morena, Maribel Luceron Lucas-Torres

**Affiliations:** 1 Health and Social Research Center, Universidad de Castilla - La Mancha, Cuenca, Spain; 2 Universidad Politécnica y Artística del Paraguay, Asunción, Paraguay; 3 Facultad de Ciencias de la Salud, Universidad Autónoma de Chile, Talca, 7500912 Chile; 4 Department of Physical Education and Health, Higher Institute of Physical Education, Universidad de la República, Rivera, Uruguay

**Keywords:** Mental quality of life, Physical quality of life, Cardiorespiratory fitness, Strength

## Abstract

**Background:**

This study aimed to examine the associations between physical fitness components and health-related quality of life (HRQoL) among adults stratified by sex and age. In addition, we aimed to examine whether these associations change based on socioeconomic, clinical, and biochemical characteristics.

**Methods:**

A total of 297 participants aged 47.41 (standard deviation: 9.08) years from the “Validity of a Model of Accelerated Vascular Aging as a Cardiovascular Risk Index in Healthy Adults: the EVasCu cross-sectional study” were included in this analysis. HRQoL, physical fitness, socioeconomic status (SES), waist circumference, and blood pressure were measured. Additionally, blood samples were extracted to determine cholesterol, triglyceride, and glycated hemoglobin A1c (HbA1c) levels. Analyses of covariance (ANCOVAs) were estimated to test mean differences in physical and mental health-related health measures (HRQoL) between fitness categories (fixed factors) by sex and age categories.

**Results:**

The physical HRQoL was related to the levels of fitness parameters among women, independent of age, while for men, it was related to better levels of general fitness and cardiorespiratory fitness among men aged < 50 and men aged ≥ 50, respectively. In contrast, mental HRQoL was related to cardiorespiratory fitness only among women aged < 50 years; speed/agility and flexibility among men aged < 50 years; and general fitness, strength, and flexibility among men aged ≥ 50 years. These data did not change when SES, clinical variables, or biochemical determinations were included in the analyses, neither for the physical nor for the mental HRQoL.

**Conclusion:**

Gender and age are important factors to be considered when analysing health indicators and influences in the population. In addition, SES, clinical characteristics, and biochemical parameters do not seem to influence the relationship between HRQoL and fitness.

**Table:** 

**Text box 1. Contributions to the literature**	
• Physical fitness throughout the lifespan and its relationship with different health parameters, including physical and mental HRQoL, have been related to health.	
• Gender and age are important factors to be considered when analysing the relationship between HRQoL and physical fitness in the middle-aged population.	
• Socioeconomic status, clinical characteristics, and biochemical parameters did not influence the relationship between HRQoL and fitness.	
• The implementation of health strategies among middle-aged adults should consider age, sex, and the relationship between HRQoL and fitness to manage patients at risk.	

## Background

The 1950s have been described as a critical time in a person’s life. Adults aged 50 years and older are at a higher risk of developing many chronic diseases that could have a negative impact on their length and quality of life, as well as on their long-term ability to live without dependency [1]. Furthermore, there are sex differences in health; men are more likely to suffer from chronic life-threatening diseases such as heart disease and cancer, while women suffer from nonfatal diseases such as autoimmune and rheumatologic diseases [2]. These differences in morbidity and mortality among men and women create a complex relationship between sex and health [3].

The health assessment included physical, mental, and social health domains, following the World Health Organization health definition [4]. In addition, subjective measurements of health and well-being have been described as being better associated with survival than objective measures are [5, 6]. In this context, the measurement of health-related quality of life (HRQoL) has been described as a valid summary of overall health and as a useful tool for estimating life-years for use in cost-effectiveness analyses [7]. Furthermore, HRQoL has been related to different health parameters across the lifespan [8], including physical fitness parameters (fitness, strength, flexibility, agility, and balance) [9]. 

Physical fitness, defined as “the ability to carry out daily tasks with vigor and alertness, without undue fatigue and with ample energy to enjoy leisure-time pursuits and to meet unforeseen emergencies” [10], decreases with age as a natural process that could hinder daily performance and compromise the capacity and function of older adults. Furthermore, low physical fitness has been related to a risk of mortality similar to smoking, hypertension, and high levels of cholesterol and is a recognized predictor of future adverse events [11, 12]. In addition, low cardiorespiratory fitness and strength have been described as risk factors for all-cause mortality [13]. Although these findings have been reported for both men and women, some sex differences in HRQoL have been related to biological and social characteristics, including different roles, social functions, and social statuses [14]. 

Due to the importance of physical fitness throughout the lifespan and its relationship with different health parameters, including physical and mental HRQoL, it seems necessary to determine how both are related at different age stages and how they could be influenced by sex and individual characteristics. Therefore, this study aimed to examine the association between physical fitness components (general fitness, fitness, strength, speed/agility, and flexibility) and HRQoL (physical and mental domains) among adults by sex and age. In addition, we aimed to examine whether these associations change based on socioeconomic, clinical, and biochemical characteristics.

## Methods

The “Validity of a Model of Accelerated Vascular Aging as a Cardiovascular Risk Index in Healthy Adults: the EVasCu cross-sectional study” collected information from healthy adults from the city of Cuenca, Spain, from June to December 2022. Participants were recruited through the distribution of flyers and posters strategically placed in public facilities, such as libraries, sports facilities, and universities. The inclusion criteria for participants were as follows: were healthy adults older than 18 years, were clinically stable in the six weeks prior to the study, and signed the informed consent form. Participants who were excluded if they were participating in another study, had diagnostic pathologies, or were receiving pharmacological treatment related to metabolic syndrome, but participants who used contraception and other unrelated treatments were included in the study. The participants who met the inclusion criteria were recruited from the facilities of the research team to undergo data collection and variable measurements on the same day.

The research protocol of this study was approved by the Clinical Research Ethics Committee of the Cuenca Health Area. (REG: 2022/PI2022). This study was designed as a cross-sectional study, and the guidelines for reporting observational studies “Strengthening the Reporting of Observational Studies in Epidemiology (STROBE) Statement” were used to conduct and report this study [14, 15]. 

### Variables

HRQoL was measured by the SF-12 questionnaire, which aimed to evaluate the intensity and/or frequency of people’s state of health. The scale is composed of twelve items that can be answered on a Likert-type scale. This questionnaire provides information on eight subscales: physical functioning, physical role, bodily pain, general health, vitality, social functioning, emotional role, and mental health. These eight subscales comprise the physical and mental domains of patients’ HRQoL; the higher the score is, the better the HRQoL. The SF-12 is a valid and reliable instrument [16]. 

Self-reported physical fitness was measured through the Spanish validated version of the International Fitness Scale (IFS) [17], which is composed of five Likert-scale questions in which the participant could rate her/his physical fitness level as ‘very poor,’ ‘poor,’ ‘average,’ ‘good,’ or ‘very good’ in comparison with the average of people of the same age. This scale includes one question about general physical fitness and four questions about its specific components (i.e., cardiorespiratory fitness, muscle strength, speed/agility, and flexibility).

Data on socioeconomic status (SES) were reported using the Spanish Epidemiology Society Scale [18], in which participants were asked to report their educational level (i.e., illiterate, no schooling, primary school, secondary school, high school, or university degree) and employment status (i.e., housekeeper, student, unemployed, employed, or freelance). In addition, marital status was self-reported and classified as single, married or cohabitant, divorced, or widowed.

Waist circumference [19] was considered the mean of three measurements using flexible tape at the midpoint between the last rib and the iliac crest at the end of a normal expiration.

Blood pressure [19] was measured using an OMRON-M5-I device (Omron Healthcare UK Ltd.). Systolic (SBP) and diastolic blood pressure (DBP) were calculated as the means of two repeated measurements, separated by 5 min each. Blood pressure was measured in a quiet place and after a 5-minute resting period using a cuff sized according to the participant’s arm circumference.

Blood samples were extracted to determine cholesterol, triglyceride, and glycated hemoglobin A1c (HbA1c) levels. Samples were extracted between 8 AM and 9 AM after 12 h of fasting. Cholesterol and triglyceride levels were determined using the Cobas 8000 Roche Diagnostics system. In addition, HbA1c was determined using the ADAMS A1c HA-8180 V analyser from A. Menarini Diagnostics®.

### Statistical analysis

The normality of the distribution of continuous variables was examined using both statistical (Kolmogorov‒Smirnov test) and graphical (normal probability plots) methods. Descriptive data are presented as the mean and standard deviation (SD) or as percentages (%). Spearman correlation coefficients for the relationships between the physical fitness parameters and the physical and mental HRQoL domains and SES (i.e., marital status, educational level, and employment status), clinical (i.e., waist circumference, SBP, and DBP), and biochemical variables (i.e., cholesterol, triglycerides, and HbA1c) were calculated by sex.

Subsequently, analyses of covariance (ANCOVAs) were performed to test the mean differences in physical and mental HRQoL scores between fitness categories (fixed factors) according to sex and age, distinguishing between participants aged 30 to < 50 years and those aged ≥ 50 years. Pairwise post hoc multiple comparisons were examined using the Bonferroni correction. For these analyses, physical fitness parameters measured with the IFIS (i.e., general fitness, cardiorespiratory fitness, muscle strength, speed/agility, and flexibility) were categorized as poor, medium, and good fitness, considering ‘very poor’ and ‘poor’ as poor fitness, ‘average’ as medium fitness, and ‘good’ and ‘very good’ as good fitness. Four different models were constructed: Model 1 was unadjusted; Model 2 was adjusted for SES, including marital status, educational level, and employment status; Model 3 was additionally adjusted for clinical variables, including waist circumference, SBP, and DBP; and Model 4 considered Model 3 and biochemical variables, including cholesterol, triglycerides, and HbA1c levels.

The statistical significance was set at *p* ≤ 0.05, and the analyses were performed using the software IBM SPSS 28 (SPSS, Inc., Chicago, IL).

## Results

The EVasCu study sample comprised a total of 406 participants. Sixteen potential participants were referred to a general practitioner and subsequently excluded from the study due to hypertension or hypercholesterolemia after the variables were measured. Among the final 390 participants, 180 women and 117 men aged ≥ 30 years were included in this analysis whose data were valid. Tables 1 and 2 present the characteristics of the included population. Gender-based significant differences were observed in clinical and biochemical variables, with cholesterol and HbA1c levels being greater among women.

**Table 1:** Characteristics of the study sample presented as mean and SD

	Women (*n*: 180)	Men (*n*:117)	
	Mean (SD)	Mean (SD)	*p*
Age (years)	48.49 (8.59)	46.50 (9.71)	0.063
Waist circumference (cm)	80.75 (12.65)	90,32 (10.75)	**< 0.001**
PAS (mmHg)	112.74 (15.59)	125.68 (13.11)	**< 0.001**
PAD (mmHg)	70.04 (10.62)	73.47 (10.01)	**0.006**
Cholesterol (mg/dl)	197.10 (35.23)	191.47 (35.36)	0.179
Triglycerides (mg/dl)	85.13 (37.25)	101.52 (65.42)	**0.006**
HbA1c (mmol/mol)	5.25 (0.31)	5.20 (0.37)	0.197
SF-12 physical	51.14 (7.72)	53.43 (5.70)	**0.006**
SF-Mental	50.43 (8.72)	52.50 (8.59)	**0.045**
Total IFIS	15.64 (3.41)	17.08 (3.38)	**< 0.001**

Notes: SD: standard deviation

**Table 2:** Categorical variables for the qualitative characteristics of the included sample as percentage

	Categories	Percentage (%)	Percentage (%)
Marital status	Single	15.6	24.1
	Married/Cohabitant	75.6	67.2
	Divorced	7.2	6.9
	Widowed	1.1	-
	Unmarried couple	0.6	1.7
Education	Primary school	1.7	0.9
	Secondary school	11.7	14.5
	High school	23.9	24.8
	University degree	62.8	59.8
Occupation	Housekeeper	4.5	1.7
	Student	2.2	1.7
	Unemployed	2.2	2.6
	Employed	81.5	78.4
	Freelance	9.6	15.5
General Fitness	Poor	13.9	5.1
	Medium	40.6	35.9
	Good	45.6	59.0
Cardiorespiratory fitness	Poor	41.1	18.8
	Medium	34.4	33.3
	Good	24.4	47.9
Strength	Poor	22.8	4.3
	Medium	46.7	43.6
	Good	30.6	52.1
Speed/agility	Poor	16.1	8.5
	Medium	50.0	43.6
	Good	33.9	47.9
Flexibility	Poor	26.1	31.6
	Medium	35.6	41.9
	Good	38.3	26.5

In this study, 180 women and 117 men aged 48.49 (SD: 8.59) and 46.50 (SD: 9.71), respectively, were included. Among women, 75.6% were married/cohabitant, 63.8% held a university degree, and 81.5% were employed. Generally, women rated their general fitness and flexibility as good (45.6% and 38.3%, respectively), while they rated their cardiorespiratory fitness as poor (41.1%) and strength and speed/agility as medium (46.7% and 50.0%, respectively). Among men, 67.27% were married/cohabitant, 59.86% held a university degree, and 78.48% were employed. Men generally rated their general fitness, cardiorespiratory fitness, strength, and speed/agility as good (59.0%, 47.9%, 52.1%, and 47.9%, respectively), while rated their flexibility as medium (41.9%).

Table 3 presents the Spearman correlations between the physical and mental domains of the HRQoL measured with the SF-12 scale; the physical fitness parameters measured with the IFIS scale; and the socioeconomic, clinical, and biochemical variables by sex. For men and women, waist circumference, cholesterol, triglyceride levels, and physical and mental HRQoL were associated with the different components of physical fitness.

**Table 3:** Pearson correlation coefficients (r) of IFIS fitness parameters with physical and mental health-related quality of life

**Men**	**General**	**Fitness**	**Strength**	**Speed/agility**	**Flexibility**
Marital status	-0.022	-0.038	0.108	-0.020	-0.003
Education	0.088	0.047	0.004	-0.089	-0.114
Occupation	-0.111	0.012	-0.034	0.011	-0.109
Waist circumference	-0.433**	-0.503**	-0.293**	-0.375**	-0.209*
PAS	-0.004	-0.068	0.038	-0.036	0.054
PAD	-0.150	-0.204**	-0.119	-0.117	-0.116
Cholesterol	-0.222*	-0.279**	-0.201*	-0.137	-0.114
Triglycerides	-0.331**	-0.391**	-0.308**	-0.35**	-0.118
HbA1c	0.079	-0.068	-0.033	-0.019	0.005
SF12-physical	0.409**	0.315**	0.196*	0.272**	0.173
SF12-mental	**0.182**	0.097	0.202*	0.218*	0.308**
**Women**	**General**	**Fitness**	**Strength**	**Speed/agility**	**Flexibility**
Marital status	-0.006	0.000	0.080	0.013	-0.062
Education	0.074	-0.011	-0.047	-0.112	0.010
Occupation	0.031	-0.045	0.047	0.036	0.053
Waist circumference	-0.331**	-0.399**	-0.167*	-0.317**	-0.366**
PAS	-0.111	-0.121	0.063	-0.148*	-0.132
PAD	-0.135	-0.131	-0.010	-0.156*	-0.162*
Cholesterol	-0.172*	-0.142	-0.154*	-0.205**	-0.144
Triglycerides	-0.272**	-0.258**	-0.131	-0.231**	-0.191*
HbA1c	-0.097	-0.010	-0.048	0.009	-0.049
SF12-physical	0.491**	0.379**	0.327**	0.340**	0.268**
SF12-mental	0.240**	0.188**	0.245**	0.214**	0.188*

ANCOVAs are presented in Table 4, considering the physical and mental domains of HRQoL as dependent variables and the physical fitness components as fixed factors. Unadjusted ANCOVA models showed that physical HRQoL was better for women reporting better levels of general fitness, cardiorespiratory fitness, strength, and speed/agility, independent of age. In addition, physical HRQoL was better among men aged < 50 years and men aged ≥ 50 years, who reported better levels of general fitness and cardiorespiratory fitness, respectively. In contrast, mental HRQoL was better among women aged < 50 years who reported better cardiorespiratory fitness; among men aged < 50 years who reported better speed/agility and flexibility; and among men aged ≥ 50 years who reported better general fitness, strength, and flexibility.

**Table 4:** Mean differences and standard error (SE) in physical and mental health-related quality of life variables by cardiorespiratory fitness levels, distinguishing by gender and age

		**Levels of fitness**							
**SF12-physical**		**Poor**		**Medium**	**Good**	**Model 1**	**Model 2**	**Model 3**	**Model 4**
General	Women (< 50)	44.36 (1.85)		49.21 (1.21)	54.85 (1.00)	**< 0.001**	**< 0.001**	**< 0.001**	**< 0.001**
	Women (> 50)	41.07 (1.97)		51.27 (1.34)	53.90 (1.12)	**< 0.001**	**< 0.001**	**0.003**	**< 0.001**
	Men (< 50)	42.71 (2.60		53.38 (1.04)	55.45 (0.77)	**< 0.001**	**< 0.001**	**0.001**	**0.003**
	Men (> 50)	50.13 (3.52)		50.18 (1.21)	54.04 (1.06)	0.058	0.094	0.042*	0.065
Fitness	Women (< 50)	48.03 (1.24)		51.95 (1.36)	54.84 (1.38)	**0.002**	**0.002**	**0.010**	**0.011**
	Women (> 50)	48.48 (1.19)		52.45 (1.30)	54.31 (1.89)	**0.015**	**0.016**	**0.048**	0.059
	Men (< 50)	50.38 (1.65)		54.11 (1.20)	55.26 (0.94)	**0.042**	**0.044**	0.415	0.193
	Men (> 50)	48.58 (1.54)		54.02 (1.35)	53.00 (1.15)	**0.029**	**0.016**	**0.018**	**0.042**
Strength	Women (< 50)	47.65 (1.74)		50.77 (1.17)	54.12 (1.30)	**0.012**	**0.014**	**0.039**	**0.018**
	Women (> 50)	47.21 (1.54)		51.14 (1.11)	54.71 (1.58)	**0.004**	**0.005**	**0.009**	**0.011**
	Men (< 50)	53.67 (2.65)		53.29 (1.08)	54.73 (0.94)	0.598	0.559	0.952	0.699
	Men (> 50)	-		50.50 (1.16)	53.92 (1.09)	**0.034**	0.053	0.110	0.349
Speed/agility	Women (< 50)	46.11 (1.95)		51.44 (1.14)	53.42 (1.26)	**0.009**	**0.006**	**0.030**	**0.036**
	Women (> 50)	45.43 (1.92)		51.09 (1.06)	53.96 (1.44)	**0.003**	**0.001**	**0.005**	**0.005**
	Men (< 50)	50.95 (2.92)		53.25 (0.99)	55.25 (0.97)	0.198	0.143	0.386	0.873
	Men (> 50)	48.78 (2.07)		51.86 (1.27)	53.67 (1.16)	0.126	0.130	0.336	0.626
Flexibility	Women (< 50)	48.01 (1.61)		51.93 (1.43)	52.74 (1.18)	0.058	0.053	0.206	0.204
	Women (> 50)	48.49 (1.52)		50.70 (1.26)	53.73 (1.46)	**0.048**	0.083	0.124	0.106
	Men (< 50)	53.22 (1.21)		53.39 (1.00)	56.49 (1.37)	0.173	0.106	0.328	0.775
	Men (> 50)	50.76 (1.39)		52.12 (1.39)	53.98 (1.43)	0.283	0.145	0.280	0.172
			**Levels of fitness**						
**SF12-mental**			**Poor**	**Medium**	**Good**	**Model 1**	**Model 2**	**Model 3**	**Model 4**
General	Women (< 50)		48.06 (1.95)	50.82 (1.28)	52.67 (1.06)	0.109	0.096	0.109	0.144
	Women (> 50)		45.94 (2.95)	47.72 (1.54)	52.41 (1.67)	0.061	0.130	0.263	0.338
	Men (< 50)		53.08 (4.61)	49.93 (1.84)	53.11 (1.36)	0.376	0.167	0.142	0.143
	Men (> 50)		56.92 (4.21)	48.04 (1.44)	57.09 (1.27)	**< 0.001**	**< 0.001**	**0.006**	**0.005**
Fitness	Women (< 50)		48.67 (1.20)	53.45 (1.31)	52.50 (1.34)	**0.019**	**0.018**	**0.026**	**0.025**
	Women (> 50)		48.76 (1.63)	48.80 (1.77)	52.09 (2.59)	0.513	0.556	0.478	0.373
	Men (< 50)		51.77 (2.68)	50.66 (1.82)	53.13 (1.52)	0.579	0.399	0.363	0.297
	Men (> 50)		50.83 (2.33)	53.23 (2.04)	54.79 (1.73)	0.402	0.547	0.877	0.978
Strength	Women (< 50)		48.84 (1.68)	51.01 (1.13)	53.18 (1.16)	0.115	0.140	0.267	0.314
	Women (> 50)		45.11 (2.08)	50.36 (1.51)	51.83 (2.13)	0.056	0.237	0.416	0.508
	Men (< 50)		51.83 (4.11)	50.16 (1.68)	53.50 (1.45)	0.329	0.290	0.206	0.294
	Men (> 50)		-	49.91 (1.48)	56.59 (1.44)	**0.002**	**0.016**	**0.011**	0.053
Speed/agility	Women (< 50)		48.58 (1.89)	50.69 (1.10)	53.32 (1.22)	0.083	0.110	0.224	0.398
	Women (> 50)		45.85 (2.64)	48.92 (1.46)	52.15 (1.98)	0.153	0.474	0.763	0.658
	Men (< 50)		38.42 (4.35)	52.13 (1.47)	53.49 (1.45)	**0.007**	**0.011**	**0.012**	**0.017**
	Men (> 50)		48.45 (2.95)	54.40 (1.81)	53.98 (1.66)	0.213	0.299	0.296	0.350
Flexibility	Women (< 50)		48.50 (1.54)	51.37 (1.37)	52.60 (1.12)	0.173	0.226	0.517	0.501
	Women (> 50)		47.28 (2.03)	48.85 (1.68)	51.97 (1.95)	0.236	0.495	0.548	0.571
	Men (< 50)		47.12 (1.81)	54.73 (1.49)	53.30 (2.05)	**0.006**	**0.006**	**0.008**	**0.009**
	Men (> 50)		48.94 (1.81)	55.61 (1.81)	55.61 (1.88)	**0.018**	0.091	0.186	**0.019**

Model1: unadjusted; Model 2: adjusted for socioeconomic status (i.e., marital status, educational level, and employment status); model 3: model 2 + clinical variables (i.e., waist circumference, SBP, and DBP); model 4: model 3 + biochemical variables (i.e., cholesterol, triglycerides, and HbA1c levels)

These data did not change when SES (i.e., marital status, educational level, and employment status), clinical variables (i.e., waist circumference, SBP, and DBP), or biochemical variables (i.e., cholesterol, triglycerides, and HbA1c) were included in the analyses (models 2, 3, and 4) for neither the physical nor the mental HRQoL.

## Discussion

This study aimed to examine the associations between physical fitness components and physical and mental HRQoL according to sex and age. In addition, we aimed to examine whether these associations change based on socioeconomic, clinical, and biochemical characteristics. Our data revealed that women reporting better physical fitness components benefitted from better physical HRQoL, independent of their age. For men aged ≥ 50 years, who reported better cardiorespiratory fitness, and for those aged < 50 years, who reported better general fitness, they enjoyed better physical HRQoL. Finally, women aged < 50 years reported better cardiorespiratory fitness, men aged < 50 years reported better speed/agility and flexibility, and men aged ≥ 50 years reported better general fitness and strength and had better mental HRQoL. These associations are reported independently of socioeconomic and clinical characteristics and biochemical determinations.

HRQoL is closely related to autoperceived physical health and reflects individuals’ personal assessment of health status and ability to perform certain activities. As it is a self-evaluation tool, the need to consider several factors, including sex, age, socioeconomic status, and clinical parameters, has been described. Among those previously mentioned, sex seems to be the most relevant factor in the relationship between fitness and physical HRQoL, regardless of age. Women are more likely to suffer from chronic diseases that could limit their participation in activities and affect their cardiorespiratory fitness and tend to report lower life satisfaction, which could affect their physical HRQoL perception [20]. It is interesting to highlight the gender differences in health when designing approaches that could help both men and women.

It has been previously reported that SES is an important factor in the assessment of HRQoL and fitness. Previous evidence has indicated that socioeconomic indices are positively associated with physical fitness, independence in daily life activities, physical functioning, and risk of chronic conditions for both genders [3, 21, 22]. A low SES is related to a greater incidence of depression and worse levels of emotional well-being among adults, with women being more likely than men to report lower physical, mental, and social health status; cognitive function; and QoL [3]. Our data confirmed that SES could influence the relationship between physical fitness and physical HRQoL, but we cannot confirm this influence on mental HRQoL. This could be due to the specific characteristics of this sample, where most of the participants were employed and married, two conditions related to better mental health [22, 23]. 

Several anthropometric variables have been previously suggested to be associated with aerobic capacity among older adults [24, 25] and have been proposed to identify adults at risk of physical limitations and multimorbidity based on age [26, 27]. A negative association between HRQoL and arterial pressure has been reported [28]. This is the first study to explore the interrelationship between these anthropometric measurements and clinical variables and HRQoL, fitness, and SES among adults. Our data indicate that SES (i.e., marital status, education, and occupation) and clinical variables (i.e., waist circumference, SBP, and DBP) are not relevant to the relationship between HRQoL and fitness level.

Ageing is accompanied by changes in body composition, biochemical parameters, and blood pressure [29, 30], and it is related to a decrease in physical fitness that could affect daily performance and, therefore [31], HRQoL. Furthermore, lower physical fitness has been related to worse levels of cholesterol and triglycerides and is a significant risk factor for all-cause mortality in both sexes [32, 33]. Blood markers have been proposed as a routine method for considering physical fitness groups in older adults [13]. Our data indicate that biochemical parameters (i.e., total cholesterol, triglyceride, and HbA1c levels) are not relevant to the association between fitness levels and HRQoL.

Cardiorespiratory fitness has been associated with an increased risk of mental health disorders in a dose‒response relationship [34], but our data suggest that differences in mental HRQoL are particularly relevant for men. Muscular fitness has been suggested to promote mental HRQoL, specifically for young men [35]. Strength is a determinant of body function that influences daily living activities and social participation among older adults and therefore could influence their HRQoL [36]. 

In addition, clinical and biochemical parameters are important variables to be considered when analysing the relationship between physical fitness and QoL in older men, as several parameters, including high cholesterol levels, are related to worse QoL [37]. The relationships between mental HRQoL and speed/agility and flexibility have been less explored, but our data show that, for young men, differences in mental HRQoL are determined by these physical fitness parameters.

This study has several limitations that should be considered. First, the participants reported their level of fitness, HRQoL, and SES. Second, the cross-sectional design of this study prevents us from establishing a causal relationship. Third, other variables that we have not considered could potentially confound the relationship between HRQoL and fitness. Fourth, the use of self-report questionnaires could introduce bias in this study, as the population may overestimate some information, including information related to physical fitness. Finally, the specific characteristics of this sample regarding occupation and marital status could bias the relationship between mental HRQoL and fitness.

## Conclusions

In conclusion, these analyses reveal new approaches for evaluating the relationship between physical and mental HRQoL and fitness. Gender and age are important factors to be considered when analysing health indicators and influences in the population. SES, clinical characteristics, and biochemical parameters did not seem to influence the relationship between HRQoL and fitness. Our data could be of interest for the analysis of health in the adult population and for the implementation of strategies to manage patients at risk.

## Data Availability

The data that support the findings of this study are available from the corresponding author, A.S.-L., upon reasonable request. **Author agreement**: Substantial contributions to the conception and design, acquisition of data, or analysis and interpretation of the data: C.A.-B., A.S.-L., E.R.-G., M.L.L.-T, and I.C.-R. Drafting the article or revising it critically for important intellectual content: C.A.-B., A.S.-L., E.R.-G., M.G.-M., B.P.-B., C.P.-M., M.L.L.-T, and I.C.-R. Final approval of the version to be published: all the authors.
